# Roles of the mitochondrial replisome in mitochondrial DNA deletion formation

**DOI:** 10.1590/1678-4685-GMB-2019-0069

**Published:** 2020-03-02

**Authors:** Marcos T. Oliveira, Carolina de Bovi Pontes, Grzegorz L. Ciesielski

**Affiliations:** ^1^ Universidade Estadual Paulista Júlio de Mesquita Filho, Faculdade de Ciências Agrárias e Veterinárias, Departamento de Tecnologia, Jaboticabal, SP, Brazil.; ^2^ Department of Chemistry, Auburn University at Montgomery, Montgomery, AL, U.S.A.

**Keywords:** Mitochondria, DNA replication, human diseases, Pol γ, Twinkle

## Abstract

Mitochondrial DNA (mtDNA) deletions are a common cause of human mitochondrial diseases. Mutations in the genes encoding components of the mitochondrial replisome, such as DNA polymerase gamma (Pol γ) and the mtDNA helicase Twinkle, have been associated with the accumulation of such deletions and the development of pathological conditions in humans. Recently, we demonstrated that changes in the level of wild-type Twinkle promote mtDNA deletions, which implies that not only mutations in, but also dysregulation of the stoichiometry between the replisome components is potentially pathogenic. The mechanism(s) by which alterations to the replisome function generate mtDNA deletions is(are) currently under debate. It is commonly accepted that stalling of the replication fork at sites likely to form secondary structures precedes the deletion formation. The secondary structural elements can be bypassed by the replication-slippage mechanism. Otherwise, stalling of the replication fork can generate single- and double-strand breaks, which can be repaired through recombination leading to the elimination of segments between the recombination sites. Here, we discuss aberrances of the replisome in the context of the two debated outcomes, and suggest new mechanistic explanations based on replication restart and template switching that could account for all the deletion types reported for patients.

## Introduction

Most animal mitochondrial DNA (mtDNA) is a compact, circular double-stranded molecule of approximately 16 kb, composed of 37 genes. Thirteen of these genes encode essential subunits of the mitochondrial respiratory chain, which in turn is responsible for the bulk of cellular ATP production via the oxidative phosphorylation (OXPHOS) process (Zeviani and Di Donato, 2004; McKinney and Oliveira, 2013). The number of mtDNA copies and the amount of mitochondria inside a cell type/tissue may vary dynamically to accommodate the cellular metabolic needs (Taylor and Turnbull, 2005). Considering the direct relationship between mtDNA copy number and the synthesis of respiratory chain subunits, the mtDNA replicative machinery, the so-called replisome, is one of the most important factors for proper maintenance of this genome and appropriate OXPHOS function.

**Figure 1:**
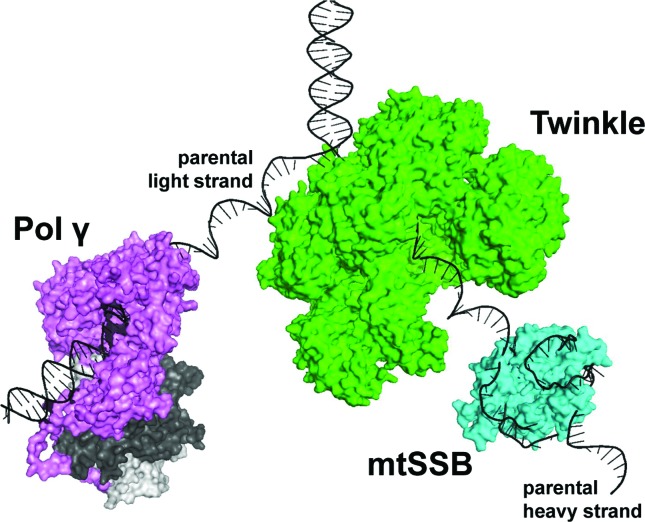
The mitochondrial replisome at a replication fork during mtDNA heavy-strand synthesis. The nuclear-encoded proteins that form the replisome are represented by the crystal structure of Pol γ (PDB: 4ZTZ), and the models of mtSSB (Oliveira *et al.*, 2011) and Twinkle (Kaguni and Oliveira, 2016). The dimeric accessory subunit of Pol γ is depicted in two tons of gray. The software Pymol (www.pymol.org) was used to analyze the structures and models and to create the figure. The scheme is not meant to detail structural and/or functional aspects of the replisome components; please see Ciesielski *et al.* (2016) for such information.

The minimum mitochondrial replisome is composed of a set of three nuclear genome-encoded proteins: the replicative mtDNA helicase Twinkle, DNA polymerase γ (Pol γ), and the mitochondrial single-stranded DNA-binding protein (mtSSB) (Figure 1) (Korhonen *et al.*, 2004; McKinney and Oliveira, 2013; Ciesielski *et al.*, 2016). During replication fork progression, the homohexameric/heptameric, ring-shaped Twinkle translocates on one DNA strand in the 5’-3’ direction, hydrolyzing nucleotide tri-phosphate and promoting the unwinding of the parental double-stranded DNA (dsDNA) (reviewed in Kaguni and Oliveira, 2016). Using the resulting single-stranded DNA (ssDNA) as template, the heterotrimeric Pol γ synthesizes a new mtDNA strand also in the 5’-3’ fashion and proofreads it using its 3’-5’ exonuclease activity. The catalytic subunit (Pol γ-α) is responsible for such activities, which are highly stimulated by its accessory Pol γ-β subunit (reviewed in Kaguni, 2004). The parental ssDNA exposed at the replication fork is protected from nucleolysis through the binding of the homotetrameric mtSSB, which also further stimulates the dsDNA unwinding by Twinkle and DNA synthesis/proofreading by Pol γ, most likely coordinating their enzymatic functions during mtDNA replication (Korhonen *et al.*, 2004; Oliveira and Kaguni, 2011).

Here, we provide a substantial review of the literature, highlighting the importance of the mitochondrial replisome functions for the mechanistic interpretations of mtDNA deletion formation, one of the most common causes of human mtDNA diseases. We discuss the clinical features of these diseases, with the support of research data from a wide range of cell culture and animal models, and reconstituted *in vitro* systems. We also provide an overview of the pathogenesis of mtDNA disorders and the molecular features of mtDNA deletions, describing previously proposed mechanisms for their formation inside mitochondria. Moreover, we present novel hypotheses that could be tested experimentally, to improve our understanding of the most abundant form of mutation in the human mitochondrial genome.

## Pathogenesis of mtDNA disorders

mtDNA diseases are metabolic disorders with an occurrence of ~1 in 5000 human individuals (Chinnery *et al.*, 2000; Elliott *et al.*, 2008). These are generally classified as primary, when arising from mutations in the mtDNA itself, or secondary, if mutations or alterations in the expression levels of nuclear genes encoding factors important for mtDNA metabolism are detected (Schon *et al.*, 2012; Alston *et al.*, 2017). The most common causes of primary mtDNA diseases are single large-scale deletions and point mutations, whereas the secondary class may arise from mtDNA depletion and multiple mtDNA deletions. Given that multiple copies of mtDNA are present in a cell, a mix of aberrant and wild-type molecules may be found in varying proportions in different tissues. This is called a heteroplasmic state, which typically does not manifest as a disease condition unless the number of mutated genomes exceed a threshold of approximately 60%. This phenomenon is tissue-dependent and some tissues may withstand higher loads of aberrant molecules (Wong, 2007; Tuppen *et al.*, 2010).

An epidemiological survey indicated that 1 in ~200 human individuals are carriers of a pathogenic mtDNA point mutation (Elliott *et al.*, 2008). Carriers of a primary, pathogenic mtDNA point mutation may remain asymptomatic for generations, up to the point in which the mutated mtDNA molecules reach the threshold level. The difference between the relatively high frequency of carriers and low frequency of diseased individuals can be explained by a balance between a genetic bottleneck and negative selection during female germ line development (for alternative possibilities, see Tuppen *et al.*, 2010; Otten *et al.*, 2018). mtDNA copy number undergoes a radical decrease in early stages of oogenesis, followed by a significant increase towards the end of the process, enabling the generation of eggs with levels of mutant mtDNA molecules higher (or lower) than in the mother’s somatic tissues. When the mutation is too severe, the eggs carrying high levels of such mtDNA are usually unviable and the mutation is often negatively selected (Fan *et al.*, 2008; Wai *et al.*, 2008). As a result, the offspring of a mother carrying a relatively mild, but yet pathogenic mutation may exhibit various levels of heteroplasmy, ranging from a virtual wild-type homoplasmy, to a predominantly aberrant haplotype with symptoms of the related mitochondrial disease (Wilson *et al.*, 2016; Alston *et al.*, 2017; Otten *et al.*, 2018). Because there is no applicable way to assess the mtDNA mutation load in maternal oocytes, a prediction of reoccurrence risk is almost impossible, although up to ~25% of pathogenic mtDNA point mutations may occur *de novo* at early developmental stages (Sallevelt *et al.*, 2017).

The primary, single large-scale deletions were the first mtDNA defects described (Holt *et al.*, 1988) and remain the most common sporadic mutations on mtDNA, accounting for approximately a quarter of all mitochondrial disorders in the human population (Schaefer *et al.*, 2008; Pitceathly *et al.*, 2012; Grady *et al.*, 2014; Gorman *et al.*, 2015). In contrast to point mutations, pathogenic mtDNA deletions typically arise *de novo* and, with rare exceptions (Chinnery *et al.*, 2004), are not inherited by the offspring (Ng and Turnbull, 2016; Kauppila *et al.*, 2017). Phenotypically, primary deletions manifest as the often-fatal Pearson syndrome in infancy, Kearns-Sayre syndrome (KSS) in childhood and adolescence, or late onset progressive external ophthalmoplegia (PEO) (Rocha *et al.*, 2018; Russell *et al.*, 2018). In the clinical scope, the mtDNA mutation load found in sporadic KSS, PEO and Pearson’s syndrome is extremely high (>80% in affected tissues) (Moraes *et al.*, 1995). This ‘clonal expansion’ of the deletion-bearing mtDNA molecules (ΔmtDNA) in affected individuals can hardly be explained by the sporadic character of the pathogenesis. In fact, a recent comparative study of the expansion of various aberrant mtDNA molecules in dividing induced pluripotent stem cells demonstrated that ΔmtDNA are preferentially replicated compared to controls or those bearing point mutations (Russell *et al.*, 2018). This finding is in line with other analyses implying that point mutation-bearing mtDNA molecules do not exhibit advantageous replication and clonal expansion (Pallotti *et al.*, 1996; Wilson *et al.*, 2016), and supports the idea of preferential expansion of ΔmtDNA (Picard *et al.*, 2016). The mechanism of clonal expansion of ΔmtDNA remains unknown (Picard *et al.*, 2016; Kowald and Kirkwood, 2018). Interestingly, the accumulation of primary ΔmtDNA has also been associated with aging of healthy human individuals (Cortopassi *et al.*, 1992; Bua *et al.*, 2006). These deletions arise often in post-mitotic tissues, such as heart, brain and skeletal muscles. However, in these cases, even though the levels of ΔmtDNA in individual cells can be very high, the overall amount of mtDNA deletions in the tissue is low, compared to pathological conditions (Cortopassi *et al.*, 1992; Schon *et al.*, 2012; Kauppila *et al.*, 2017).

Secondary mtDNA diseases result from mutations in nuclear genes and their inheritance follows the Mendelian pattern, often in an autosomal dominant fashion (Ng and Turnbull, 2016; Alston *et al.*, 2017). The major causes of secondary mtDNA diseases identified to date involve defects in the mitochondrial replisome: mutations in the *POLG1* and *TWNK* genes, which encode respectively Pol γ-α and Twinkle (Van Goethem *et al.*, 2001; Copeland, 2014; Nurminen *et al.*, 2017; Rahman and Copeland, 2019). Pathogenic mutations in genes related to other mitochondrial processes, such as OXPHOS, fusion and fission, intra-mitochondrial translation, etc., have also been found and are reviewed elsewhere (Ng and Turnbull, 2016; Alston *et al.*, 2017). To date, mutations in >250 nuclear genes encoding mitochondrial proteins have been related to mitochondrial disorders in general (Mayr *et al.*, 2015).

The most common consequence of defects in the mitochondrial replisome is the accumulation of multiple large-scale deletions in mtDNA (Rahman and Copeland, 2019), which are formatively similar to the primary single large-scale deletions described above (Reeve *et al.*, 2008; Schon *et al.*, 2012). Furthermore, multiple large-scale deletions generated by an aberrant mitochondrial replisome are most commonly associated with myopathies and PEO in adult patients, which may also be manifest in the primary syndromes such as PEO and KSS (Van Goethem *et al.*, 2001; Agostino *et al.*, 2003; Di Fonzo *et al.*, 2003; Filosto *et al.*, 2003; Wanrooij *et al.*, 2004; González-Vioque *et al.*, 2006; Lehmann *et al.*, 2016; Grier *et al.*, 2018). These similarities may suggest that both primary and secondary mtDNA deletions may emerge upon similar shortcomings of the mtDNA replication machinery. In addition, a recent comparative study demonstrated that ΔmtDNA molecules generated in patients with *POLG1* mutations undergo clonal expansion similarly to the single large deletions sporadically generated in patients. In contrast, ΔmtDNA generated in patients with a mutation in *OPA1*, which encodes a protein involved in mitochondrial fusion, did not expand clonally (Trifunov *et al.*, 2018). In both *POLG1*-related and sporadic deletions (but not in *OPA1*-related deletions), the authors observed an increase in the overall mtDNA copy number, which may link the phenomenon of clonal expansion to a compensatory upregulation of replication as a response to inefficient OXPHOS.

In contrast to primary mtDNA diseases, defects of the mitochondrial replisome may lead to different, frequently coexisting, mtDNA aberrances: point mutations are often found along with mtDNA deletions in the same patient/tissue. A mouse line expressing a proofreading-deficient Pol γ-α variant, the so-called mutator mouse, may serve as an important model for the understanding of this phenomenon, as the accumulation of multiple large-scale deletions in its mtDNA has been observed (Trifunovic *et al.*, 2004; Fuke *et al.*, 2014), in addition to the expected accumulation of point mutations (Vanderstraeten *et al.*, 1998; Spelbrink *et al.*, 2000). Despite a previously considered possibility that the accumulation of point mutations may predispose mtDNA molecules to deletions, no correlation has been found between the distribution of point mutations and the deletion breakpoints (Wanrooij *et al.*, 2004; Hudson and Chinnery, 2006). Furthermore, heterozygous mice encoding the proofreading-deficient Pol γ-α on a single allele do not accumulate point mutations (they may happen, but are likely corrected by the product of the wild-type *POLG1* allele), but still develop large-scale deletions (Fuke *et al.*, 2014). The parallel occurrence of mtDNA point mutations and deletions may suggest that the proofreading activity might not be the most critical function of the exonuclease domain of Pol γ-α, whose active site was abolished in the mutator mouse (Nurminen *et al.*, 2017). In support, some mutations in the exonuclease domain result in decreased nucleotide polymerization rather than defects in proofreading (Szczepanowska and Foury, 2010), and the exonucleolytic activity by Pol γ is necessary for the *in vitro* production of ligatable 5’ ends for proper mtDNA replication (Macao *et al.*, 2015).

mtDNA depletion is relatively rare and typically causes early-onset mitochondrial diseases (Moraes *et al.*, 1991; Suomalainen and Isohanni, 2010). Although the inefficient replication of mtDNA is an obvious cause, the exact mechanism(s) remain(s) unexplained. Intriguingly, *POLG1* mutations that cause point mutations and deletions in the mtDNA can also cause mtDNA depletion when the individual carries two mutated, pathogenic alleles. Moreover, the same *POLG1* mutations can cause early onset encephalopathy with severe mtDNA depletion or late-onset PEO with ataxia (Horvath *et al.*, 2006; Nguyen *et al.*, 2006; Tzoulis *et al.*, 2006). Therefore, mtDNA depletion syndromes appear to result from similar insufficiencies of the mitochondrial replisome as other late-onset secondary mitochondrial diseases. However, more studies are necessary to fully understand the pathogenesis of the depletion syndromes.

## Formation of mtDNA deletions

Since KSS was first reported by Kearns and Sayre (1958), there have been many reports describing deletions of different sizes and at different mtDNA positions among patients (Holt *et al.*, 1988; Nelson *et al.*, 1989; Montiel-Sosa *et al.*, 2013; Damas *et al.*, 2014; Saldaña-Martínez *et al.*, 2019). The most common pathogenic large-scale deletion of 4977 bp (the so-called `common deletion’) is precisely flanked by perfect direct repeats (DR) of 13 bp, at nucleotide positions 13,447–13,459 (within the *ND5* gene) at the 5’ end, and at positions 8470–8482 (within the *ATPase8* gene) at the 3’ end (Schon *et al.*, 1989; Samuels *et al.*, 2004). As a result, the genes for two complex V subunits, one complex IV subunit, four complex I subunits and five tRNAs are lost. Approximately 60% of mtDNA deletions reported to date are similarly flanked by perfect DR sequences; these are called class I deletions. Of the remaining, 30% are flanked by imperfect repeats (class II deletions) and 10% have no flanking repeats (class III deletions) (Mita *et al.*, 1990; Samuels *et al.*, 2004; Reeve *et al.*, 2008; Chen *et al.*, 2011). The secondary, multiple large-scale deletions generated due to mutated *POLG1* or *TWNK* bear characteristics of the class II deletions because of the imperfect flanking repeats observed (Zeviani *et al.*, 1989; Wanrooij *et al.*, 2004).

**Figure 2:**
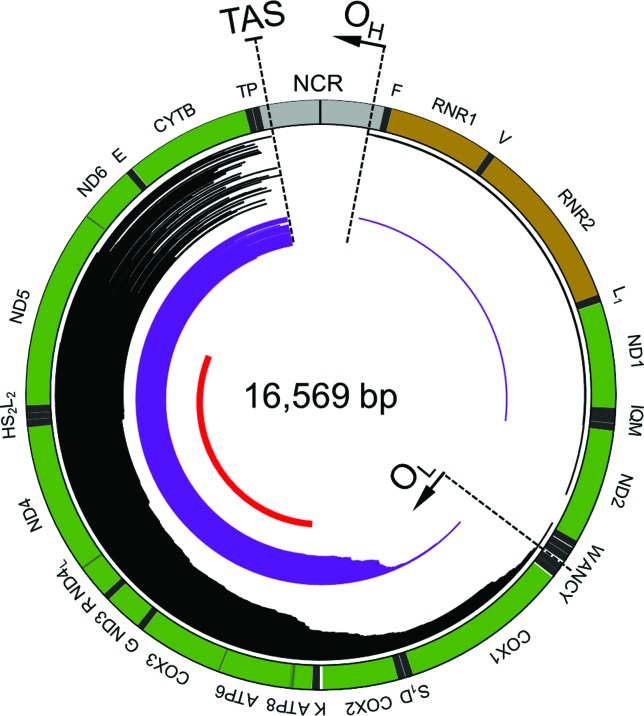
Diagram of the distribution of selected deletions within the human mitochondrial genome. The location of the deletions corresponds to the distribution of lines inside the mtDNA ideogram. Each line represents a single deletion. The location of 152 reported single mtDNA deletions is presented as black lines. The location of 86 reported multiple mtDNA deletions associated with progressive external ophthalmoplegia (PEO), including those generated upon mutations in *POLG1* and *TWNK*, is presented as light blue lines. The location of the common deletion is presented as the red line. Origins of replication O_H_ and O_L_, as well as the termination associated sequence (TAS) are labeled. Protein-coding genes are colored in green; ribosomal RNAs are colored in brown; transfer RNAs are colored in grey; the non-coding region (NCR) is colored in light grey. The diagram was generated with and modified from the mitochondrial DNA breakpoints database, MitoBreak (Damas *et al.*, 2014).

**Figure 3:**
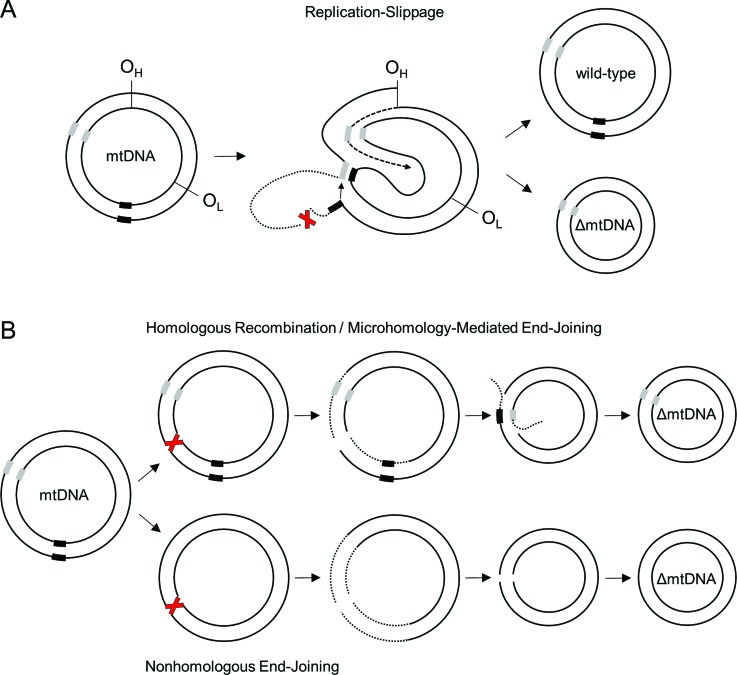
Schematic representation of the debated mechanisms of mitochondrial DNA deletion formation. (A) In the replication-slippage (i.e. slipped mispairing) mechanism, a break of the displaced heavy DNA strand (indicated with a red X), generated during an asynchronous replication process, enables pairing of the 3’ flanking region of that heavy-strand (grey box) with the 5’ flanking region of the light-strand (black box), ahead of the leading DNA synthesis (dashed arrow). The resulting unpaired DNA flap (dotted line) is then degraded and ligated (solid arrow) with the remainder of the heavy-strand. Replication initiated from O_L_ would yield a daughter mtDNA molecule carrying the deletion of a segment ahead of the 3’ flanking region. The scheme represents the model proposed by Shoffner *et al.* (1989), and it was modified to incorporate the location of the heavy-strand breaks according to the results reported in Phillips *et al.* (2017). The position of the flanking regions corresponds to the 3’ and 5’ direct repeats (DRs) of the common deletion. (B) The formation of deletions in the homologous recombination- or microhomology-mediated end-joining-based mechanism (upper panel) is initiated by a double strand break (indicated with a red X), which occurs predominantly within the region between the two replication origins. Excision of both heavy and light-strands in 3’-5’ direction (dotted lines) enables pairing of the unveiled complementary flanking regions (grey and black boxes). Next, flap excision and subsequent ligation would generate a daughter mtDNA molecule carrying the deletion and retaining one flanking region. The scheme represents the model proposed by Krishnan *et al.* (2008). Class III mtDNA deletions have been suggested to form via nonhomologous end-joining of linear molecules (lower panel), which to date remain uncharacterized. The scheme has been modified from Chen *et al.* (2011).

Notably, the vast majority of all deletions occurs between the mtDNA sites O_H_ and O_L_ (Figure 2) (Reeve *et al.*, 2008; Damas *et al.*, 2014; Belmonte *et al.*, 2016), recognized as origins of the heavy- and the light-strand replication, respectively (vertebrate mtDNA strands are denoted as heavy and light due to their distinct nucleotide composition; for details, see Ciesielski *et al.*, 2016). This striking accumulation of deletions between the two replication origins suggests that their formation mechanism is related to the replication process (Tuppen *et al.*, 2010). According to the strand-displacement model of mtDNA replication, synthesis of the new heavy- (leading) strand initiates at O_H_ and proceeds using the light-strand as template. The displaced parental heavy-strand remains single-stranded, coated by mtSSB or hybridized to RNA. After synthesis reaches approximately two-thirds of the mtDNA circumference, the replisome unveils O_L_ on the parental heavy-strand, enabling replication of the new light- (lagging) strand to initiate at this site by a new replisome and proceed in the opposite direction (see the extensive reviews of current models of mtDNA replication by Holt and Jacobs, 2014; Ciesielski *et al.*, 2016). The first model of mtDNA deletion formation was proposed upon the analysis of the common deletion (class I) and is based on the replication-slippage mechanism (Shoffner *et al.*, 1989). In this scenario, incidental breaks of the displaced parental heavy-strand between the 3’ and 5’ DR enable annealing of the 3’ DR from that strand to the distant complimentary 5’ DR on the parental light-strand, brought to proximity by structural rearrangements during synthesis of the nascent heavy-strand (Shoffner *et al.*, 1989; Mita *et al.*, 1990). Subsequent synthesis from O_L_ would omit the stretch between the break point and the paired repeats, generating a deletion (Figure 3A). This mechanism has been found especially appealing in early reports indicating that the mtDNA molecules bearing the common deletion retain the 3’ DR but not the 5’ DR, which indeed fits the model (Shoffner *et al.*, 1989; Degoul *et al.*, 1991; Chen *et al.*, 2011). In support, a recent elegant report using mito-TALENS in human cultured cells determined that nicks in the heavy-strand, in the vicinity of the 5’ DR are sufficient and necessary to yield the common deletion (Phillips *et al.*, 2017). *In vivo*, reactive oxygen species (ROS) could pose as the potential source of damage that triggers the DR mispairing. Replicating mtDNA molecules are associated with the inner mitochondrial membrane, frequently in close proximity to the OXPHOS complexes (Rajala *et al.*, 2014). Hence, mtDNA can be permanently exposed to ROS, with well-established detrimental effects (Shokolenko *et al.*, 2009).

Even though the replication-slippage mechanism could explain the class I deletions, it hardly explains the formation of class II and III deletions, which constitute approximately one third of the reported cases (Mita *et al.*, 1990; Degoul *et al.*, 1991). An alternative was proposed by Schon and colleagues, who based on combined analyses of cases of class I (including the common deletion), II and III deletions, suggested that recombination may underlie their formation (Schon *et al.*, 1989; Mita *et al.*, 1990). This idea was further developed into a model that assumed that cross-pairing of DR in the formation of class I deletions, or imperfect repeats in the formation of class II deletions, would facilitate efficient homologous recombination or microhomology-mediated end-joining (MMEJ) events, which, similarly to the replication-slippage mechanism, would yield a copy of a single flanking sequence in the daughter ΔmtDNA (Krishnan *et al.*, 2008; Tadi *et al.*, 2016) (Figure 3B). A later study on multiple large-scale deletions in patients with mutated *POLG1* or *TWNK* indicated that double-stranded breaks (DSBs) could be involved in the formation of ΔmtDNA (Wanrooij *et al.*, 2004). Homologous recombination is one of the fundamental DSB repair mechanisms in a wide spectrum of genetic systems, which made the recombination-mediated mechanism of ΔmtDNA formation plausible, although historically lack of recombination has been erroneously associated with animal mtDNA (Harrison, 1989; Avise, 2000). The model was further supported by circumstantial evidence for recombination in mitochondria, including the concurrent accumulation of linear and ΔmtDNA forms, the observation of four-way junctions, catenates and other recombination intermediates, the identification of specific nuclear recombination factors in mitochondria, and others (Srivastava and Moraes, 2005; Bacman *et al.*, 2009; de Souza-Pinto *et al.*, 2009; Fukui and Moraes, 2009; Pohjoismäki *et al.*, 2009,^ 2011^; Sage *et al.*, 2010; Ciesielski *et al.*, 2018). Perhaps the most compelling evidence comes from a study that demonstrated that mitochondrial protein extracts from distinct rat tissues and from HeLa cells were able to mediate joining of DNA substrates bearing microhomologies between 5 and 22-nt, which are similar to the flanking regions of the class I and II deletions (Tadi *et al.*, 2016). Additionally, nonhomologous end-joining (Figure 3B) (Lieber, 2010), which would account for the formation of the least frequent class III deletions (Srivastava and Moraes, 2005; Fukui and Moraes, 2009; Nissanka *et al.*, 2018), has been clearly reported for the yeast *Saccharomyces cerevisiae* (Kalifa *et al.*, 2012), but substantial evidence from mitochondria of humans or animal models is yet to be delivered (Fishel *et al.*, 2007; Tadi *et al.*, 2016; Nissanka *et al.*, 2018).

A shortcoming of the proposed DSB repair-mediated mechanism of deletion formation, however, lies in recently published data demonstrating that linearized mtDNA molecules have a very short half-life in cells, being rapidly degraded by unknown nucleases, with no evidence for any robust DSB repair process (Moretton *et al.*, 2017). Efficient elimination of linearized mtDNA molecules, as a result of DSBs induced by targeted enzymatic cleavage of mutant mtDNA in patient-derived cells, has been previously shown to reduce heteroplasmy by allowing repopulation of the organelles with the undigested wild-type mtDNA molecules (Bacman *et al.*, 2013; Gammage *et al.*, 2014). Moraes and colleagues have recently discovered that this rapid degradation of linear mtDNA molecules depends on the exonucleolytic activity of Pol γ (but not on its polymerase activity) and that large deletions of mtDNA are accumulated in the exonuclease-deficient Pol γ mice (Nissanka *et al.*, 2018), which is in line with the earlier reports on the mutator mouse (Trifunovic *et al.*, 2004; Fuke *et al.*, 2014). These results imply that the DSB-induced degradation of linearized molecules and recombinational repair are actually competing/complementing processes, with a significant advantage for the former. Earlier reports did in fact indicate low levels of DSB repair in mitochondria (Bacman *et al.*, 2009; Fukui and Moraes, 2009). Overall, the correlation between the frequency of DSBs and deletion formation in mitochondria appears to be well documented (Fukui and Moraes, 2009; Ciesielski *et al.*, 2018; Nissanka *et al.*, 2018), and in the light of these reports, including our own studies, they are very likely related. Perhaps, the coexistence of the rapid degradation of linearized mtDNA and the low efficiency of DSB repair could actually explain the clonal expansion phenomenon, as the rapid degradation of many cleaved molecules (which could arise in abundance, for example, from uncoupling of the mitochondrial replisome, as discussed in the next section) would give advantage to the replication of those few repaired ΔmtDNA, which consequently would repopulate mitochondria.

In the putative DSB repair mechanism, the resulting free ends are susceptible to being resected completely by 3’-5’ exonucleases, allowing the homologous (or micro-homologous) repeats to pair. The resulting overhangs are exposed to degradation, so subsequent ligation of the nicks on both strands would generate circular ΔmtDNA capable of being replicated (Figure 3B) (Krishnan *et al.*, 2008). Pol γ is the only 3’-5’ exonuclease in mitochondria, hence it is a likely candidate to promote the putative resection of DSB-afflicted mtDNA molecules. Notably, Kunz and coworkers have recently proposed an appealing model for linear mtDNA degradation, in which Pol γ acts in concert with another mitochondrial exonuclease, MGME1, to degrade both DNA strands simultaneously, according to their substrate preferences, i.e. 3’-5’ for Pol γ, and 5’-3’ for MGME1 (Kornblum *et al.*, 2013; Peeva *et al.*, 2018). However, other studies indicate that the role of MGME1 is secondary, if not dispensable (Moretton *et al.*, 2017; Nissanka *et al.*, 2018). Perhaps, MGME1 action fluctuates depending on the physiological context: when functional, MGME1 and Pol γ would degrade linear mtDNA fragments; if MGME1 is unfunctional or absent, Pol γ alone would generate overhangs, which could later hybridize and serve as material for deletion formation. Markedly, MGME1 knockout results in the accumulation of mtDNA deletions in mice (Matic *et al.*, 2018), which is consistent with the idea of higher frequency of overhangs in the absence of MGME1. Other enzymes necessary for the putative maturation of DSB-repaired mtDNA are also present in mitochondria. Fen1 and Dna2 are nucleases specific to the processing of 5’-DNA flaps (Zheng *et al.*, 2008; Kazak *et al.*, 2013). Recently, the activity of mitochondrial ligase III, Lig3, has been demonstrated to be important for the MMEJ in mitochondrial extracts (Tadi *et al.*, 2016), and along with MGME1 and Dna2, it appears to be necessary for the formation of the common deletion in cultured cells (Phillips *et al.*, 2017). However, the same study also implicated the mitochondrial replisome components (Pol γ, Twinkle, and mtSSB) in the formation of the common deletion, which underscores the role of the replication process in deletion formation or indicates new roles for the replication proteins in DSB repair that are yet to be described. Nevertheless, it seems that the suite of proteins necessary to mediate DSB repair by recombination is present in human mitochondria.

It has been suggested in several reports that both mechanisms, the replication-slippage and the DSB repair, may coexist and facilitate the formation of distinct deletion classes; e.g., replication-slippage would account for the class I deletions, whereas DSB repair would lead to classes II and III, or, alternatively, that replication-slippage may be specific to sporadic deletions, and DSB repair to the secondary deletions (Degoul *et al.*, 1991; Mita *et al.*, 1990; Wanrooij *et al.*, 2004; Tuppen *et al.*, 2010; Chen *et al.*, 2011; Ciesielski *et al.*, 2018). However, comparative studies of all three classes of deletions revealed remarkable similarities in the distribution of deletion breakpoints, which were independent of the presence of repeat sequences (Samuels *et al.*, 2004; Reeve *et al.*, 2008; Guo *et al.*, 2010). These analyses imply that classifying the deletions in three distinct groups on the basis of sequence homology may be somewhat misleading, and that the same principal mechanisms operate in the formation of almost all mtDNA deletions.

## The role of the replisome in the formation of mtDNA deletions

One major difference between the two models of deletion formation lies in the role of the mitochondrial replisome proteins. According to the early replication-slippage mechanism, deletions are technically formed during the replication process, hence the activity of the replisome is critical (Shoffner *et al.*, 1989). In the DSB repair-related model, deletions are formed in an independent process of recombination/recircularization, by a yet uncharacterized machinery that facilitates one of the proposed recombination types (Krishnan *et al.*, 2008; Chen, 2013). Importantly, the latter scenario received significant recognition upon observations that DSBs and the formation of deletions correlate with the stalling of the replication fork (Wanrooij *et al.*, 2004,^2007^; Pohjoismäki *et al.*, 2011). Correspondingly, some studies indicate that, while the distribution of the 5’ deletion breakpoints is more variable, the 3’ breakpoints predominantly localize in the vicinity of the termination associated sequence (TAS) site at the end of the D-loop structure (Zeviani *et al.*, 1989; Samuels *et al.*, 2004; Wanrooij *et al.*, 2004; Damas *et al.*, 2014; Jemt *et al.*, 2015) (Figure 2). TAS is the prominent replication arrest site even under normal physiological conditions: ~90% of all replication events initiated at O_H_ terminate there, yielding the 7S DNA and the characteristic triplex structure of the D-loop region (Doda *et al.*, 1981; Bowmaker *et al.*, 2003; Ciesielski *et al.*, 2016). This co-localization of the prominent replication arrest site and the 3’ breakpoints of deletions indicates that replication failure precludes the formation of deletions.

In agreement with these observations, studies of the biochemical properties of pathogenic mitochondrial replisome components linked their dysfunctions with replication stalling. The most commonly reported pathogenic mutation of *POLG1*, the A467T replacement (Chan and Copeland, 2009; Nurminen *et al.*, 2017), compromises the Pol γ holoenzyme formation (i.e., the interactions between Pol γ-α and the accessory Pol γ-β) and results in reduction of the polymerase activity and stalling during DNA synthesis *in vitro* (Chan *et al.*, 2005). Affected patients accumulate deletions but manifest variable clinical symptoms (Rajakulendran *et al.*, 2016; Nurminen *et al.*, 2017). In addition, all known pathogenic mutations in the *POLG2* gene (which encodes Pol γ-β) affect Pol γ holoenzyme assembly and stability, which as for *POLG1* A467T, results in the accumulation of ΔmtDNA (Young *et al.*, 2011; Copeland, 2014). *In vivo* and *in vitro* studies of error-prone Pol γ variants, such as *POLG1* Y995C or the earlier discussed exonuclease-deficient variant, indicated that these also stall frequently during DNA synthesis and lead to the accumulation of multiple deletions (Wanrooij *et al.*, 2004, 2007; Szczepanowska and Foury, 2010; Song *et al.*, 2011). Notably, it is currently considered that the lower fidelity of these Pol γ variants results in mechanistic shortcomings, which could also underlie the formation of deletions (Chan and Copeland, 2009; Szczepanowska and Foury, 2010; Nurminen *et al.*, 2017).

Mutations in *TWNK* that alter the biochemical activities of Twinkle result in the accumulation of multiple large-scale deletions in mtDNA and the development of PEO (Suomalainen *et al.*, 1997; Spelbrink *et al.*, 2001; Tyynismaa *et al.*, 2005; Copeland, 2014). The characterization of mtDNA replication intermediates (RIs) using bidimensional-agarose gel electrophoresis (for the detailed experimental procedure see Reyes *et al.*, 2007, 2009) in cells expressing pathogenic helicase variants indicated multiple replication stalling events and frequent DSBs (Wanrooij *et al.*, 2007; Goffart *et al.*, 2009; Pohjoismäki *et al.*, 2011). Intriguingly, overexpression of defective Twinkle in mice (creating the so-called “deletor mouse” line) resulted in less severe symptoms as compared to the mutator mouse (Tyynismaa *et al.*, 2005), perhaps because the endogenous wild-type *TWKN* locus is still intact in the deletor mouse. Even though this mouse model accumulates multiple deletions, it exhibits relatively good physical performance and no traits of accelerated aging, which are the hallmarks of the mutator mouse (Trifunovic *et al.*, 2004). The major difference between the two mouse models is the accumulation of point mutations by the mutator mouse. Interestingly, a heterozygous mouse, expressing the proofreading-deficient Pol γ from a single allele, does not exhibit point mutation accumulation, as mentioned earlier, nor the progeroid phenotype, but like the deletor mouse, bears deletions and symptoms typical of PEO (Fuke *et al.*, 2014). These results indicate that mtDNA deletions (including the primary single large-scale deletions) result in similar phenotypic effects, which implies a uniform mechanism for their formation. In the case of Pol γ defects, however, the co-accumulation of point mutations may further alter/complicate clinical symptoms, which could explain the larger clinical spectrum of *POLG1*-disorders (Rahman and Copeland, 2019). In the case of Twinkle dysfunctions, mtDNA replication stalling in cultured human cells and in tissues of six-week-old deletor mice is evident, although the former never accumulates deletions, and the latter only shows ΔmtDNA much later in life (Goffart *et al.*, 2009). These findings indicate that the events that lead to mtDNA deletion in post-mitotic tissues can be modelled in proliferating cells, again pointing to a common mechanism of deletion formation irrespective of tissue or cell type.

Stalling of the replication fork may directly result from mtDNA secondary structures, which are resolved during normal replication, but pose hurdles for a defective replisome. Such idea has been proposed to explain class I deletions, as their flanking regions have high potential to form hairpin-like secondary structures due to sequence complementarity (Mita *et al.*, 1990; Damas *et al.*, 2012). The difficulty of applying this explanation to class II and III deletions, which share micro- to no complementary flanking regions, drove scientists to consider other recombination-based scenarios (Mita *et al.*, 1990; Krishnan *et al.*, 2008). However, computational analyses of mtDNA sequences indicated that the general distribution of the deletion breakpoints overlaps with the distribution of sites with high potential to form guanine-quadruplex (GQ) structures (Oliveira *et al.*, 2013; Bharti *et al.*, 2014; Dong *et al.*, 2014). These are non-B DNA secondary structures characterized by planar stacks of guanines interacting by nonconventional Hoogsteen hydrogen bonds (Hou and Wei, 1996; Bharti *et al.*, 2014; Dong *et al.*, 2014). In the nucleus, GQs have been demonstrated to hinder DNA replication and cause genome instability (Ribeyre *et al.*, 2009; Lopes *et al.*, 2011). Furthermore, sequences with GQ-forming potential have been associated with deletions and duplications in the genomic DNA of cancer cells (Ribeyre *et al.*, 2009; De and Michor, 2011). Recently, GQs within human mtDNA have been observed directly using fluorescence microscopy (Huang *et al.*, 2015), and computational analyses have associated GQs with the formation of sporadic and secondary mtDNA deletions (Dong *et al.*, 2014). Regarding the common deletion, both the 5’ and the 3’ 13 bp repeats have the potential to form GQs in three overlapping configurations (Oliveira *et al.*, 2013; Dong *et al.*, 2014). Markedly, a single nucleotide polymorphism within the 5’ repeat, 8472C>T, abolishes the guanine run shared by all three possible GQs and, consequently, the formation of the common deletion is significantly reduced in the haplogroup bearing such substitution (whereas the distribution of other deletions seems to be unaltered) (Guo *et al.*, 2010). The TAS region has earlier been predicted to form stem-loop structures (Doda *et al.*, 1981; Brown *et al.*, 1986; Pereira *et al.*, 2008), however, it also has the potential to form a GQ structure (Dong *et al.*, 2014). Importantly, the GQ-forming potential does not exclude the formation of other secondary structures, such as hairpin-, cruciform- and cloverleaf-like (Damas *et al.*, 2012). In fact, in their comprehensive study on the GQ-forming potential of mtDNA, Kaufman and coworkers have also confirmed the potential for the formation of other secondary structures facilitated by direct repeats (Dong *et al.*, 2014). Perhaps an additive potential for the formation of GQs and other secondary structures may explain the higher frequency of deletions facilitated by direct repeats (class I). Direct evidence for the relevance of mtDNA secondary structures for ΔmtDNA formation comes from studies on the Pif1 helicase, which catalyzes the resolution of GQs and other secondary structures in the nucleus and in mitochondria (Paeschke *et al.*, 2013; Mendoza *et al.*, 2016). Pif1 ablation in mice increases the mtDNA deletion load in skeletal muscles (Bannwarth *et al.*, 2016), and its depletion in human cell lines results in a 2-fold increase in the common deletion formation in response to TALEN-induced breaks of the mtDNA heavy-strand (Phillips *et al.*, 2017).

**Figure 4:**
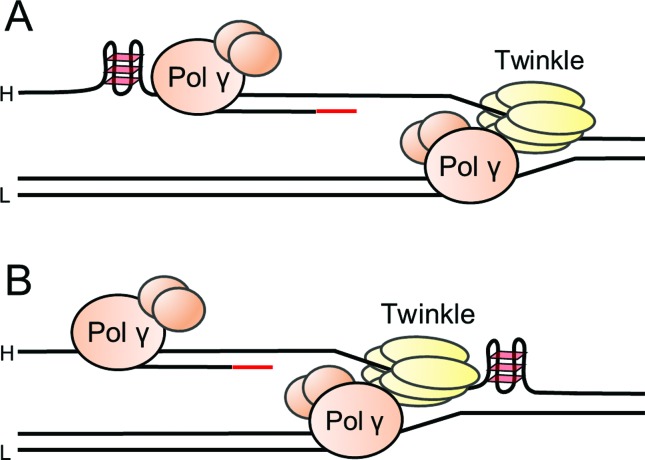
Schematic representation of potential stalling of a mitochondrial replisome at G-quadruplex structures (GQs) formed on the heavy-strand template (denoted as H). GQs (shown as three rhomboids) pose as a potential structural obstacle for the lagging-strand synthesis by Pol γ on the heavy-strand template (A), or for the Twinkle helicase during leading-strand synthesis on the light-strand template (denoted as L) (B). The red line represents the RNA primer generated at O_L_. Alternative priming sites generated by PrimPol on the heavy-strand template (not depicted here), permit passing the obstacles and would explain the “rescue replication”, as suggested by Torregrosa-Munumer *et al.* (2017). See text for more details.

Nearly all putative GQs are predicted to form on the heavy-strand, which is consistent with its high G-content (Oliveira *et al.*, 2013; Dong *et al.*, 2014). Considering the strand-displacement replication model, secondary structures on the heavy-strand would primarily affect the synthesis of the nascent light-strand. Notably, studies on mtDNA RIs indicated that, in comparison to the heavy-strand synthesis, the light-strand synthesis is significantly delayed and exhibits higher susceptibility to halting in the presence of dideoxynucleotides (Wanrooij *et al.*, 2007). Moreover, Falkenberg and coworkers demonstrated directly that subnormal levels of Pol γ-α cause inefficient replication of the nascent light-strand (with unaltered replication of the nascent heavy-strand), and result in mtDNA deletions and PEO in patients (Roos *et al.*, 2013). Stalling of Pol γ on the parental heavy-strand would generate breaks consistent with the replication-slippage model (see above, Figure 4A). Also, secondary structures inherent to the heavy-strand (formed before another replication round) would pose a hurdle for the helicase, which operates on such a strand (Figure 4B). In fact, Brosh and coworkers demonstrated that *in vitro* the wild-type Twinkle is incapable of resolving intra-strand GQs (Bharti *et al.*, 2014). Additionally, analysis of mtDNA RIs in cells expressing defective Twinkle variants indicated that replication is abolished due to arrest in the vicinity of TAS (Wanrooij *et al.*, 2007), which is consistent with the observed mtDNA copy number decrease in associated cases. Hence, the replicative helicase is highly vulnerable to secondary structures formed on the heavy-strand, to the extent of halting of the processive replication.

In striking consistency with the accumulation of GQs on the heavy-strand, Sfeir and coworkers recently reported that not only double-strand breaks, but “just a nick” in the heavy-strand in proximity to the 5’ breakpoint is sufficient to generate the common deletion in mtDNA molecules of human cells in culture (Phillips *et al.*, 2017). Furthermore, the authors of this study demonstrated that the formation of the common deletion requires the presence of Pol γ, Twinkle and mtSSB, as their depletion resulted in a dramatic drop in ΔmtDNA upon induced cleavage. This study advocates that the replisome components are involved in the formation of deletions and/or that replication and deletion formation are somehow linked. This is somewhat contradictory to the fact that a dysfunction of the replisome (either by the knockdown of one of its component or by the presence of a mutated constituent) may also result in deletions of all classes (Reeve *et al.*, 2008). In our opinion, this inconsistency could be explained by the existence of a threshold effect for replisome activity, above which defective replisomes retain competence for genome replication (but may also facilitate the formation of replication-related deletions); below this threshold, replication halts resulting in mtDNA depletion (which in fact occurs in early-onset syndromes). In support of such a threshold hypothesis, a decrease in the levels of Pol γ-α transcripts by half in a heterozygous *POLG1* knockout mouse does not affect its development, whereas the homozygous *POLG1* knockout is lethal at the early embryonic stage (Hance *et al.*, 2005). This lack of an ‘intermediate’ phenotype implies significant tolerance to the limited level/activity of Pol γ. Also, the phenomenon of clonal expansion seems to be consistent with the concept of threshold activity, as the lack of replication capacity would also exclude any genome expansion, and yet it has recently been documented for deletions resulting from defective Pol γ (Trifunov *et al.*, 2018). Therefore, each individual who develops beyond the embryonic stage must possess the capacity to replicate mtDNA. A direct implication of this hypothesis is that deletion formation would not be dictated by the replication capacity, but rather by the frequency of which the deletion-forming mechanism is “triggered”, i.e. the frequency of heavy-strand breaks, which would also determine the severity of the resulting condition. A defective replisome could induce such breaks in every replication round explaining why mutations in *TWNK* and *POLG1* are the most common causes of mtDNA diseases. Interestingly, in contrast to a defective Pol γ, the RIs resulting from a defective Twinkle that stalls before reaching O_L_, appear to be fully dsDNA (Wanrooij *et al.*, 2007). This might suggest that cells expressing defective Twinkle could have a lower frequency of single-strand breaks in the heavy-strand, as these would be prevented *in vivo* by annealing to a second strand of DNA (e.g., a nascent strand, product of replication) or of RNA (as product of the RITOLS/bootlace process). On the other hand, the observation that fully double-stranded RIs appear before the fork reaches O_L_ implies that the stalling of Twinkle may initiate light-strand synthesis independently of O_L_. This finds support in earlier reports that suggested the existence of alternative light-strand replication initiation sites (Brown *et al.*, 2005), as well as in a recent report demonstrating that Twinkle may promote the initiation of replication at sites distinct from the two origins (Cluett *et al.*, 2018). Pohjoismaki and coworkers recently demonstrated that such advantageous replication mechanism does in fact exist and can rescue the stalled mtDNA replication (Torregrosa-Muñumer *et al.*, 2017). This `rescue replication’ involves the cooperative action of Pol γ and PrimPol, which is an RNA/DNA polymerase also found in the nucleus, capable of laying primers on the mtDNA to be utilized by Pol γ (García-Gómez *et al.*, 2013; Stojkovic *et al.*, 2016; Xu *et al.*, 2019). The activity of such an alternative replication mechanism could explain the less deleterious symptoms associated with the deletor mouse, specifically the lack of motor function impairment, which is present in the exonuclease-deficient Pol γ mice.

Stalling of the replication fork appears to be a common consequence of various defects to the replisome components that precludes heavy-stand breaks, which directly triggers the formation of deletions. However, in the case of primary deletions, the source of heavy-strand breaks remains puzzling, as all the replisome components are supposedly functional. As mentioned earlier, Sfeir and coworkers proposed that the breaks could result from ROS activity. However, this view is currently debated as there is no clear association of elevated ROS and accumulation of ΔmtDNA (Nissanka *et al.*, 2018), and neither the mutator nor the deletor mice exhibit elevated ROS (Trifunovic *et al.*, 2005; Tyynismaa *et al.*, 2005; Edgar *et al.*, 2009). Another possibility considered by Sfeir and coworkers was an uncontrolled activity of DNA metabolic enzymes. In agreement with this idea, in our recent study on mtDNA replication in *Drosophila melanogaster*, we observed that an elevated expression of wild-type Twinkle resulted in the accumulation of ΔmtDNA, which expanded clonally to the extent that no wild-type mtDNA was detected by Southern-blotting (Ciesielski *et al.*, 2018). Deletions have also been reported for mice overexpressing the wild-type helicase (Ylikallio *et al.*, 2010). Although there are structural and functional differences between the fly and mammalian Twinkle and Pol γ homologues (Stiban *et al.*, 2014; Ciesielski *et al.*, 2015; Oliveira *et al.*, 2015), these observations prompted us to speculate that not only mutated replisome components but also dysregulation of their levels, in a way that would change their stoichiometry at the replication fork, is deleterious and may result in mtDNA deletion formation. Importantly, overexpression of Twinkle in our study, as well as in the recent study by Holt and coworkers (Cluett *et al.*, 2018), resulted in the accumulation of RIs consistent with replisome stalling. This observation strongly suggests that stalling of the replication fork is a universal step in the formation of deletions in both primary and secondary syndromes.

Markedly, the effects of overexpressing Twinkle in fly cells resemble the effects of halting Pol γ in human cells with dideoxynucleotides, to which the mitochondrial replicase is highly sensitive. In both cases, RIs are consistent with replication stalling and exhibit long stretches of ssDNA, as well as substantial incorporation of RNA (Wanrooij *et al.*, 2007; Ciesielski *et al.*, 2018). These features are consistent with desynchronization between unwinding of the template DNA and DNA synthesis *per se*. The milestone report on the human minimal mitochondrial replisome implied that effective DNA synthesis in the “mini-circle” assays requires functional interaction between both enzymes (Korhonen *et al.*, 2004). In support, it has recently been demonstrated that a specific pathogenic mutation in human *POLG1* does not alter the biochemical properties of the enzyme, but interrupts its functional interaction with Twinkle (Qian *et al.*, 2015). Therefore, it is highly likely (although still speculative) that during processive DNA synthesis, the mitochondrial replisome, similarly to many other replication systems, acts as a complex containing the two enzymes and, perhaps mtSSB (Lee and Richardson, 2011), at least for the mtDNA heavy-strand synthesis. It seems likely that uncoupling the activity of both enzymes (e.g. upon overactivity of the helicase or halting of Pol γ) results in the generation of single-stranded stretches ahead of the polymerase, vulnerable to breaks. In fact, we observed that Twinkle overexpression in fly cells results in a 50% increase in the abundance of linear mtDNA molecules (Ciesielski *et al.*, 2018). Interestingly, dideoxynucleotide-restriction of Pol γ activity generates the same pattern of RIs as defective Pol γ variants bearing human mutations, an observation that is supportive of the presumed uniform mechanism of deletion formation (Wanrooij *et al.*, 2007). Considering the possibility of mitochondrial replisome uncoupling, one would need to address the etiology of such an event in healthy tissues before trying to understand disease and aging. In this notion, certain xenobiotics, such as berberine, a plant alkaloid used in the treatment of diabetes and other conditions (Kong *et al.*, 2004; Cicero and Baggioni, 2016), possess GQ-stabilizing properties and accumulate in mitochondria (Pereira *et al.*, 2007; Bazzicalupi *et al.*, 2013). Accumulation of such agents could potentially stall the activity of a fully functional mitochondrial replisome (Naeem *et al.*, 2018), which poses as an appealing circumstantial cause for the formation of primary mtDNA deletions, especially those accumulating with aging. This possibility warrants further investigation.

Stalling of the replication fork often results in single- or double-strand breaks (reviewed in Mirkin and Mirkin, 2007; Cannan and Pederson, 2016). The mechanism by which a stalled replication fork generates breaks on mtDNA remains elusive. Generally, single-stranded DNA is more vulnerable to breaks, and double-strand breaks are a consequence of unrepaired single-strand breaks in a double-stranded DNA molecule. The heavy-strand displaced during mtDNA replication is therefore the “weak side”, as the light-strand remains double-stranded. This is consistent with the idea that heavy-strand breaks is the major trigger for mtDNA deletion formation, as discussed above. Oxidation inflicted by ROS is often considered a direct break-inducing damage, however, as mentioned earlier, this possibility is currently under debate. Alternatively, breaks could result from the tension imposed on mtDNA by the progressing replisome, which usually results in positive supercoiling ahead of the fork. If unresolved, superhelical strains may be relieved by inter-winding of strands behind the fork, which in the case of a single-stranded molecule, could result in breaks in a phosphodiester bond. We find this possibility especially interesting, as it could also result in the formation of mtDNA catenanes (if no break occurs), which is another hallmark of Twinkle overexpression that we and others have observed (Pohjoismäki *et al.*, 2009; Ciesielski *et al.*, 2018). Nevertheless, the link between replication fork stalling and mtDNA breaks warrants further experimental evidence.

## What exactly happens after the break?

**Figure 5:**
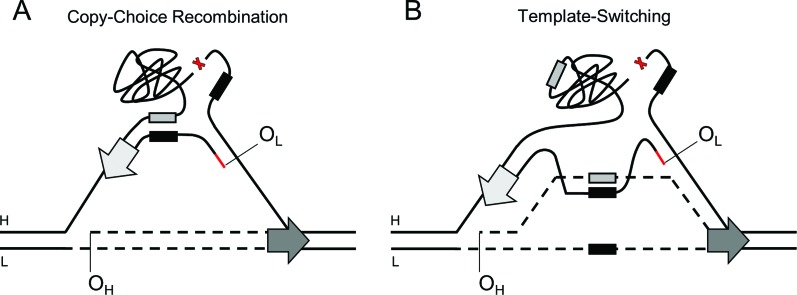
Schematic representation of the mechanisms of mtDNA deletion formation via copy-choice recombination (A) or template-switching (B). In both scenarios, DNA synthesis of the light-strand initiated at O_L_, using the heavy-strand template (denoted as H), is stalled after the 5’ deletion’s flanking region (black box), which promotes template breaking (indicated with a red X) between the deletion flanking regions. The 5’ flanking region of the nascent light-strand may pair with the complementary 3’ flanking region of the parental heavy-strand (gray box), which would enable replication restart (A). Alternatively, the nascent light-strand may invade the nascent heavy-strand (which remains paired with the parental light-strand, denoted as L; both strands represented as dashed lines), and pair with its complementary 3’ flanking region (gray box). Such a duplex structure would serve as a transient primed-template for DNA synthesis, until the damage site is bypassed and replication can resume on the parental heavy-strand (B). The block arrows represent the replisome progressing in the indicated directions during heavy- (dark gray) and light- (light gray) strand syntheses. The red line represents an RNA primer generated at O_L_.

Although replication stalling is acknowledged as an important culprit behind pathological mtDNA deletions, almost nothing is known about the fate of stalled replication intermediates, such as their subsequent processing, and which enzymatic players are involved. The replication-slippage model proposed earlier is based in essence on the concept of ‘slipped mispairing’ of direct repeats between both DNA strands (Shoffner *et al.*, 1989). The shortcoming of this scenario is that for the slipped mispairing to occur, the heavy-strand would need to be displaced ahead of the fork during leading-strand synthesis (Figure 3A), for which there is no evidence during the regular replication process. Interestingly, such RIs would be generated upon the hypothesized replisome uncoupling. However, taking the evidence that replication stalling during mtDNA light-strand synthesis underlies the formation of deletions, another possibility needs to be considered: that the mispairing does not have to occur between the two parental strands, but may in fact take place between the parental heavy-strand and the nascent light-strand. In such a case, a replisome stalled on, for example, a GQ site during light-strand synthesis, downstream of the 5’ DR (of the common deletion), would generate a break like those induced with mito-TALENS. The nascent light-strand containing a copy of the 5’ DR could potentially anneal to the 3’ DR of the parental heavy-strand (Figure 5A). This would create a new primed template sufficient for replication restart. The daughter, deletion-bearing mtDNA molecules would be consistent with those observed in class I deletions (and others), i.e., retaining the 3’ flanking region. Such mechanism of the replication-slippage model (known specifically as copy-choice recombination) has been proposed upon intensive studies on prokaryotic and viral replication systems (Viguera *et al.*, 2001, and references therein). Notably, both Pol γ and Twinkle share viral ancestry (Oliveira *et al.*, 2015; Kaguni and Oliveira, 2016), and the copy-choice recombination mechanism agrees with major findings regarding the formation of mtDNA deletions: i) this mechanism depends on replication stalling on the heavy-strand template; ii) the mispairing of flanking sites could occur independently of the heavy-strand replication, which resolves the issue of the annealing of parental strands; iii) repair of the nicked parental heavy-strand would require exonuclease and ligase activities, consistent with the reported importance of MGME1, Dna2 and Lig3 for the formation of the common deletion (Phillips *et al.*, 2017); and iv) no specialized recombination machinery is required, consistent again with the report by Sfeir and coworkers. Remarkably, as this work was under review, a report was published providing strong *in vitro* evidence for the copy-choice recombination mechanism (Persson *et al.*, 2019), which occurs in a way very much similar to our predictions (Figure 5A).

Interestingly, Pol γ, like other polymerases, is tolerant to a certain level of primer-template mismatch, as long as the 3’ end is complementary (Bebenek and Kunkel, 2000; Longley *et al.*, 2001). Perhaps, this could explain the class II and III deletions based on imperfect repeats and micro- to no homologies, and the higher frequency of the class I deletions, which would be based on the high complementarity of the flanking regions. Additionally, utilization of a mismatched primer-template substrate by Pol γ could potentially give rise to point mutations, which as mentioned above, are frequently found concomitantly to deletions, although no correlation between the distributions of deletion breakpoints and point mutations in the mtDNA has been detected to date (Wanrooij *et al.*, 2004).

Alternatively, replication stalling on the heavy-strand template would also enable the nascent light-strand to invade and pair with the nascent heavy-strand, serving as a “bypass” template to overcome a stalling site. This possibility is consistent with the strand-displacement model in which the nascent heavy-strand would be laid on the light-strand template before the initiation of DNA synthesis at the O_L_. After bypassing the structural hurdle, replication would continue back on the heavy-strand template. This so-called template-switching mechanism (Figure 5B) is well documented in various organisms (Giannattasio *et al.*, 2014; Lovett, 2017), and could take place in mitochondria by the recently reported strand-exchange activity of Twinkle (Sen *et al.*, 2016). Template-switching typically results in the accumulation of catenated circular DNA molecules (Giannattasio *et al.*, 2014), which was in fact observed along with mtDNA deletions in our study on the effects of Twinkle overexpression in fly cells (Ciesielski *et al.*, 2018). A similar phenotype was observed in human heart muscle, as well as upon Twinkle overexpression in mice heart, brain, and skeletal muscle (Pohjoismäki *et al.*, 2009). Since mtDNA replication and deletion formation could be dependent on the mitochondrial nucleoid context, which still needs to be better understood especially in different post-mitotic tissues, it is possible that both copy-choice recombination and template-switching play a role *in vivo*, although this is highly speculative at this point.

In our opinion, the results of the studies presented in this review strongly support the replication-based models of mtDNA deletion formation, which is initiated upon replication stalling on and possibly breaks of the heavy-strand template. This promotes mispairing of the nascent light-strand with complementary sites on another accessible DNA strand. Resolution of the slipped-template may require a simple replication restart or a template-switching mechanism. We believe that the proposed mechanism accommodates all the major findings in the field. Notably, this replication-mediated recombination model does not exclude the existence of independent DSB-repair systems in mitochondria, such as homologous recombination or non-homologous end-joining. However, further studies are warranted to provide strong biochemical evidence for such processes, including the complete identification of their protein players and the associated enzymatic activities.
